# The immune cell landscape and response of Marek’s disease resistant and susceptible chickens infected with Marek’s disease virus

**DOI:** 10.1038/s41598-023-32308-x

**Published:** 2023-04-01

**Authors:** Wesley C. Warren, Edward S. Rice, Ashley Meyer, Cari J. Hearn, Alec Steep, Henry D. Hunt, Melissa S. Monson, Susan J. Lamont, Hans H. Cheng

**Affiliations:** 1 Department of Animal Sciences, University of Missouri, Columbia, MO USA; 2 Avian Disease and Oncology Laboratory, USDA, ARS, USNPRC, East Lansing, MI USA; 3 Department of Human Genetics Program, University of Michigan Medical School, Ann Arbor, MI USA; 4 Department of Animal Science, Iowa State University, Ames, IA USA; 5 Food Safety and Enteric Pathogens Research Unit, USDA, ARS, NADC, Ames, IA USA

**Keywords:** Computational biology and bioinformatics, Genetics, Immunology

## Abstract

Genetically resistant or susceptible chickens to Marek’s disease (MD) have been widely used models to identify the molecular determinants of these phenotypes. However, these prior studies lacked the basic identification and understanding of immune cell types that could be translated toward improved MD control. To gain insights into specific immune cell types and their responses to Marek’s disease virus (MDV) infection, we used single-cell RNA sequencing (scRNAseq) on splenic cells from MD resistant and susceptible birds. In total, 14,378 cells formed clusters that identified various immune cell types. Lymphocytes, specifically T cell subtypes, were the most abundant with significant proportional changes in some subtypes upon infection. The largest number of differentially expressed genes (DEG) response was seen in granulocytes, while macrophage DEGs differed in directionality by subtype and line. Among the most DEG in almost all immune cell types were granzyme and granulysin, both associated with cell-perforating processes. Protein interactive network analyses revealed multiple overlapping canonical pathways within both lymphoid and myeloid cell lineages. This initial estimation of the chicken immune cell type landscape and its accompanying response will greatly aid efforts in identifying specific cell types and improving our knowledge of host response to viral infection.

## Introduction

Marek’s disease virus (MDV), a highly oncogenic alphaherpesvirus that infects chickens, causes great losses to the poultry industry by inducing T cell lymphomas and immunosuppression in susceptible birds^1,2^. MDV establishes persistent infection in its host with clinical symptoms as early as three weeks post infection. Vaccines against Marek’s disease (MD) are routinely administered to all commercial poultry but have repeatedly lost efficacy over time due to the evolution of new and more virulent MDV strains. While MD vaccines are highly protective in controlling tumor incidence, it is hypothesized that their inability to eliminate viral replication or transmission has been a major factor in the emergence of more virulent MDV strains in MD-vaccinated flocks^2^.

MDV evades the host immune response through inhibition of important pathways, such as downregulation of MHC class I^3^, thus, allowing the virus to achieve latency and establish persistent lifelong infection. MDV can escape innate immunity through downregulation of interferon expression^4^; multiple MDV proteins, including the viral oncogene, *Meq*, are able to inhibit type 1 interferon production via the cGAS-STING pathway, which may allow evasion of early innate responses to viral infection, and escape of antitumor pathways^5^. MDV genes can also undergo alternative splicing in infected B cells, suggesting ongoing viral diversity is a key part to its survival in the host^6^. The dynamics of MDV emergence are correlated with numerous factors such as bird age, time of year, host genetic background, and diet, making intervention and control very challenging^7,8^.

The search for a better understanding of MD resistance is complicated by its polygenic basis with the major histocompatibility complex (MHC) locus as a known major influence on MD resistance^1^ as well as non-MHC genetic factors. Avian Disease and Oncology Laboratory (ADOL) lines 6_3_ and 7_2_ are two highly inbred White Leghorn lines that are relatively MD resistant and susceptible, respectively^1^, and have proven invaluable for understanding the underlying mechanisms of genetic resistance to MD. Hereafter, these lines are referred to as resistant and susceptible. Because both lines share the same B2 MHC haplotype, this greatly enables identification of non-MHC genes that influence MD incidence. Despite the complexity of MD resistance, researchers have identified a large number of candidate genes that collectively account for over 80% of the genetic variance between the two lines with allele-specific expression differences observed in response to MDV infection within and between lines^9^. Also, a GWAS study of MD resistance has identified 38 QTLs that after retesting the underlying variants in elite commercial lines suggests many of these loci are associated with overcoming MDV infection^10^. These genetic studies collectively show the expected polygenic nature of the MD genetic resistance.

Transcriptome analysis in MDV infection research has thus far either involved the preparation of bulk RNA from whole organ homogenates^11^ or the isolation and study of a few single cell types, such as macrophages^12^. The host response to MDV within macrophages from both MD resistant and susceptible lines showed significant gene regulatory changes with stronger virally induced responses in cells from susceptible birds (n = 1,265 genes regulated)^12^. Among lymphocytes, B cells are infected early following MDV entry into the lung and a large (n = 2,186) in vitro DEG response was found in MDV-infected B cells compared to control B cells, with an enrichment for cytokine-cytokine receptor activation pathways^13^. While these studies have yielded gene expression signals relevant to the host immune response to MDV infection in specific cell types or as aggregate transcriptomes, the context of coregulation of molecular pathways by all cell types has been missing. Cell type specific responses to systemic infection are important for understanding MDV’s evasion of immune response, and single-cell RNA sequencing (scRNAseq) now allows for reconstruction of distinct cell populations and their gene expression patterns. Recent work broadly illustrates the depth of exploration possible when investigating immune response by cell type regardless of the virus type or host species^14,15^.

**Figure 1:**
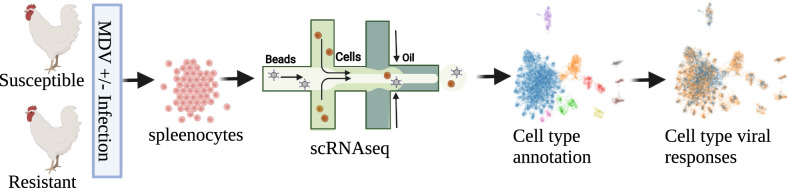
Experimental design to differentiate splenic-derived lymphocyte transcriptome responses to MDV infection in resistant and susceptible lines of chickens.

In this study, we performed scRNAseq to determine the splenic cellular reaction during the cytolytic phase (six days post infection (dpi)) in MD resistant or susceptible chickens when in uninfected control or MDV-infected states (Fig. 1). Our study adds to the prior but limited immune cell landscape knowledge of the chicken^16,17^, especially in spleen, using scRNAseq of splenic-derived mononuclear leukocytes; we annotated major cell populations based on known chicken immune cell markers and inferred similarities to mammalian immune cell types. We then determined the compositional and transcriptional changes occurring upon MDV infection for these immune cell types*.* The use of birds infected with virulent MDV and uninfected controls from both resistant and susceptible lines served to identify differences in early cellular responses, which are likely to be important for early control of viral replication and tumorigenesis.

## Results

### Cellular transcriptomics

Our previous work with these two lines demonstrated robust viral responses in measured phenotypes of resistance and their gene expression^1,9^. For this study birds by line at one week of age were intra-abdominally infected or not and then evaluated in the later part of the acute phase. After splenic-derived leukocyte harvest from MDV-infected and control birds from both lines, we recovered 22,566 cells after standard quality control filtering (see methods). In total, 14,378 cells passed further quality control metrics for gene number expressed, total read counts, and percent counts mapping to mitochondrial and ribosomal genes. A median of 478 genes were detected per cell. To account for both batch effects and true differences between cells of the same type in different samples, we integrated cells across samples using Harmony^18^. We manually inspected all initial clusters after dimensionality reduction using uniform manifold and approximation projection (UMAP^19^) and unsupervised clustering of all cellular transcriptomes using the Leiden algorithm^20^. The distribution of individual cells by cluster are mostly uniform by viral infection state or across genetic lines (Suppl. Figure 1) to yield 12 transcriptionally distinct clusters of cells for further analysis.

### Cell type identification

**Figure 2:**
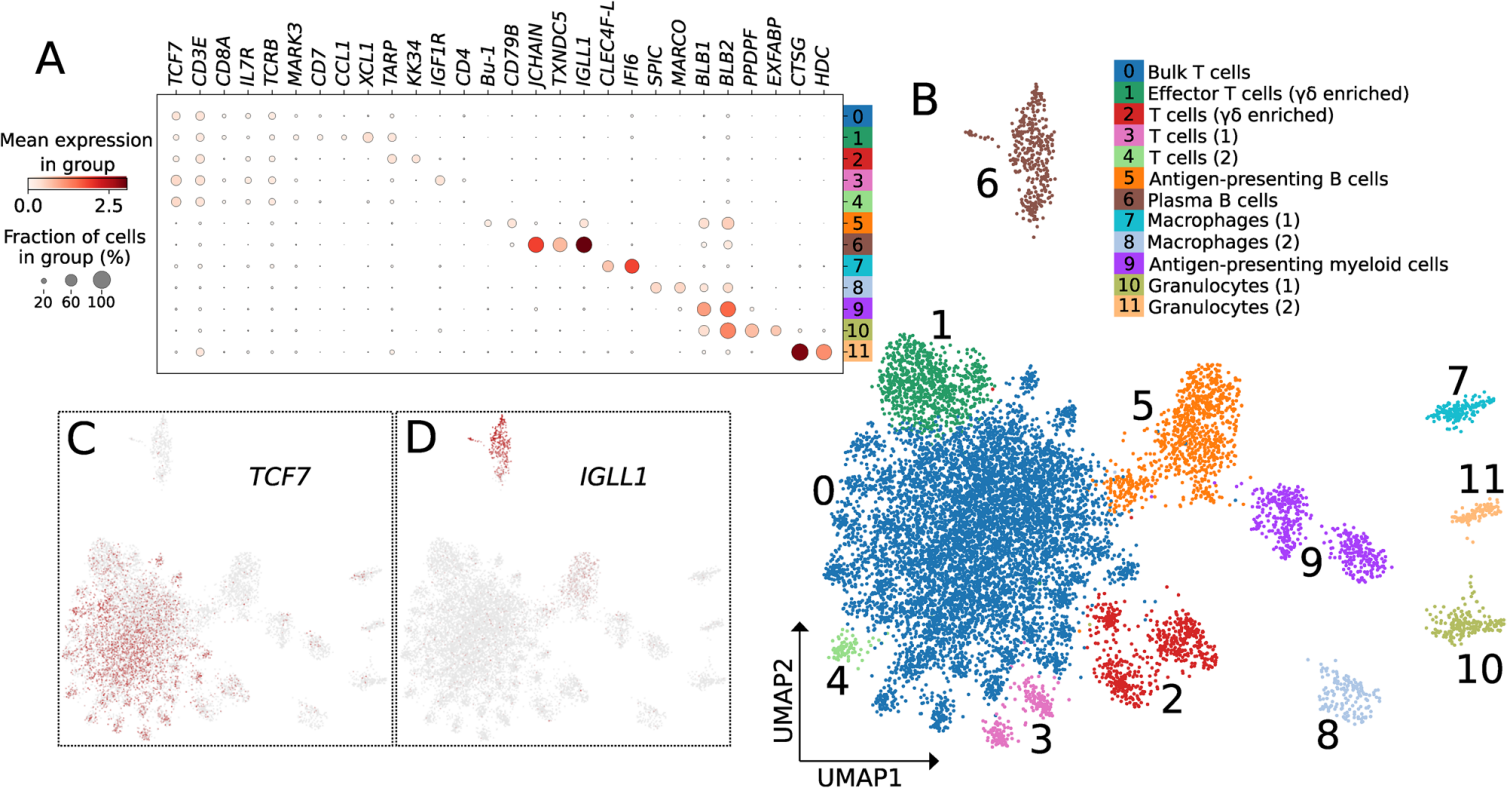
Cell clusters and assigned identities. (**A**) Cluster-specific expression of immune cell marker genes used to assign cell identities to the 12 clusters. Percentage of cells in a cluster expressing a gene (dot size) and mean expression intensity (dot shade) are both represented in this plot. We used these data to assign a cell identity to each numbered cluster, labeled on the right and corresponding to cluster colors in (**B**). (**B**) Uniform manifold approximation and projection (UMAP) plot of all immune cells across samples and conditions. Cells are partitioned into 12 Leiden clusters, numbered and colored based on cluster assignment. (**C**,**D**) UMAP plots with each cell colored by its level of expression of *TCF7* (**C**) and *IGLL1* (**D**), demonstrating that some markers are expressed by all subtypes of a more general cell type, such as *TCF7*, which is expressed by all Tcell subtypes (**C**), whereas other markers are specific to a single cluster, such as *IGLL1*, which is primarily expressed by plasma B cells, with lower expression in antigen-presenting B cells (**D**).

Before cell type identification, DEGs in each cluster compared to all other clusters were identified. As there are few well-curated chicken gene markers for major immune cell types, manual curation was essential, which included inference with human and mouse scRNAseq databases in combination with searches of chicken immune studies for avian-specific expression patterns. Some DEGs in each cluster were unannotated (i.e., just Ensembl gene identifiers), so additional curation was carried out to assign gene ontology, if possible. For example, upon further curation the unannotated *ENSGAL00000015461* gene is *Bu-1*, a definitive marker of chicken B cells^21^. However, multiple DEGs had neither orthologs with other species nor previous studies determining their ontology (Suppl. Table 1). Our identification of the cell type mostly used the top 20 significantly DEGs per cluster (Suppl. Table 1; *p* < 0.01; Fig. 2A), where our assignments represent a prediction based on cells that occupy a transcriptionally distinct cluster. Future experiments should validate if these varied immune cell types truly define a cell type, e.g., antigen presenting or plasma B cells. Some genes demonstrate the shared lineage expected from prior studies, e.g., transcription factor 7 (*TCF7*; Fig. 2C), an established T cell marker, while others are more cell-type specific, e.g., Ig λ chain (*IGLL1*; *ENSGALG00000049450*; Fig. 2D) in plasma B cells. We hereafter report our cell type predictions by their lymphoid or myeloid categorizations (see Fig. 2B).

### Lymphoid cells

Cluster 0, the largest grouping, appears to be an aggregate of T cell types (Fig. 2B), enriched for αβ T cells with the presence of T cell receptor (TCR) β chain (*TCRB*; *ENSGALG00000014754*; Fig. 2A; Suppl. Figure 2). Some cells belonging to this cluster also expressed *TARP* (TCR γ chain; Fig. 2A; Suppl. Figure 2), but αβ T cells are the most abundant. T cell gene markers *TCF7* (Fig. 2C), CD3ε molecule (*CD3E),* CD4 molecule (*CD4*), CD8α molecule (*CD8A)*, and interleukin 7 receptor (*IL7R)* are also present (Suppl. Table 1). Due to limited resolution, we were not able to further subdivide this cluster into sub-clusters representing these constituent Tcell subtypes. Although there is a clear signal showing a group of CD4-expressing T cells inside the "bulk T cell" cluster, we were unable to adjust clustering parameters in a way that separated these cells from the other T cells in this cluster in our analysis. Therefore, we labelled cluster 0 as bulk T cells.

Cluster 1 showed γδ T cell enrichment based on *TARP* (Fig. 2A) and TCR δ chain (*TCRD*; *ENSGALG00000043654*; Suppl. Table 1) expression. T cell lineage genes such *CD7* and interleukin 2 receptor subunit beta (*IL2RB)* were expressed (Fig. 2A; Suppl. Table 1) and when considering the localized expression of X-C motif chemokine ligand 1 (*XCL1)*, a chemokine associated with T cell activation^22^ (Fig. 2A; Suppl. Figure 2), we classified cluster 1 as effector T cells (γδ enriched).

In cluster 2, *TARP* and *TCRD* expression are also observed (Fig. 2A; Suppl. Table 1) but compared to effector T cells (γδ enriched), we suggest cluster 2 cells are in a different state due to *GM-CSF* family chicken-specific cytokine *KK34* gene expression (Fig. 2A; Suppl. Figure 2). Another distinguishing T cell marker was the retinoic acid related orphan receptor (*RORC*; Suppl. Table 1) that is a therapeutic target for T cell-associated diseases in humans^23^. *RORC* (in its γt isoform) is strongly associated with TH17 immune responses in mammals, including classical TH17 helper T cells, certain TH17-like innate lymphocytes, and γδ T cell populations ^23,24^. This cell type may represent chicken TH17-like T cells; however, further characterization is needed to demonstrate typical TH17 cytokine activity. We therefore labelled cluster 2 as T cells (γδ enriched).

Clusters 3 and 4 expressed T cell markers but less definitive evidence was available to assign specific T cell type (Suppl. Table 1). In cluster 3, *TCF7, IL7R*, *CD3E,* and insulin like growth factor 1 receptor *(IGF1R*) indicate assignment of a general T cells (1) label was appropriate (Fig. 2A; Supp. Table 1). Cluster 4 was similarly designated at T cells (2), due to the expression of T cell lineage markers, such as *TCF7*, GRB related adaptor protein 2 (*GRAP2*), and CD3ζ molecule (*CD3Z*, also known as *CD247;* Fig. 2A,C).

Clusters 5 and 6 were predicted to contain B lymphocytes that we categorized as antigen-presenting and plasma B cells, respectively. Cluster 5 expressed the B cell markers *Bu-1*, a B cell marker in chickens^21^, and CD79B molecule (*CD79B;* Fig. 2A; Suppl. Figure 2), the signaling chain of the B cell receptor (BCR) complex. Annotation of cluster 6 was based on the marker genes Ig λ chain *(IGLL1*), IgM (*JCHAIN*), and thioredoxin domain containing 5 (*TXNDC5;* Fig. 2A,D; Suppl. Figure 2). High *TXNDC5* expression suggest cluster 6 represents a plasmablast/plasma cell state.

### Myeloid cells

Among possible myeloid cell types, we identified antigen-presenting cells (cluster 9), granulocytes (clusters 10 and 11), and two macrophage types (clusters 7 and 8; Fig. 2B). Cluster 9 contains cells involved in antigen processing and presentation as suggested by strong expression of MHC class II beta chain genes^25^ (*BLB1* and *BLB2*; Fig. 2A). Cluster 9 also expresses the Fc receptor gamma subunit (*FCER1G*; Suppl. Table 1)*,* consistent with an antigen-presenting myeloid cell label.

In cluster 10, extracellular fatty acid-binding protein (*EXFABP*) and pancreatic progenitor cell differentiation and proliferation factor (*PPDPF;* Suppl. Figure 2) expression suggests annotation as granulocyte (1). In chicken spleen, *EXFABP* is overexpressed in various myeloid cell types when challenged by *Salmonella*^26^, and is constitutively expressed in heterophils^27^ while *PPDPF* in humans is enriched in eosinophils relative to other immune cell populations^28^. We predict cluster 11 to be another granulocyte subtype, which we labelled as granulocytes (2); this is supported by high expression of cathepsin-G (*CTSG*), which is expressed in the granules of chicken heterophils^27^ and histidine decarboxylase (*HDC*), associated with basophil or mast cell-like populations^29^ (Fig. 2A).

Cluster 7, labelled as macrophages (1), expressed macrophage markers interferon alpha inducible protein 6 (*IFI6*) and C-type lectin domain family 4 member F (*CLEC4F-like*; Fig. 2A). Additionally, this cluster expressed both subunits (*ITGA2B* and *ITGB3*) of the CD41/CD61 integrin alpha IIb/beta III complex, which is a known thrombocyte marker in the chicken^30^; thus, this population may also overlap thrombocytes, which in the chicken are nucleated immune cells known to share response pathways with myeloid cell populations^31^. Cluster 8 expressed macrophage markers Spi-C transcription factor (*SPIC*) and macrophage receptor with collagenous structure (*MARCO*) that led us to label them macrophages (2) (Fig. 2A)*.* Additionally, cluster 8 expressed C-X-C motif chemokine ligand 13 (*CXCL13*), a B cell chemoattract *in vitro*^32^. These two macrophage cell types expressed very few of the same genes that justified their separate identities (Suppl. Table 1). However, further categorization of splenic macrophage cell types is needed as various spleen macrophage and dendritic cell populations can be distinguished by other genes including *CSFR1* and *CD11c*^33^.

### Expression of viral transcripts

**Figure 3:**
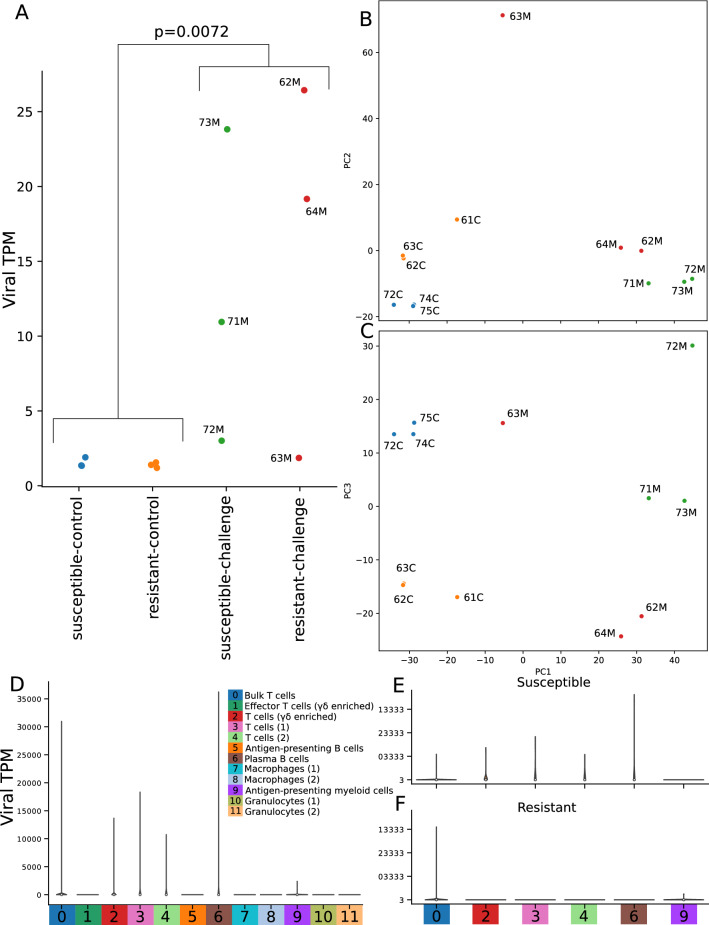
Viral transcription by individual, challenge status, genetic line, and cell type. (**A**) Viral transcripts per million in each library, separated by line and challenge status. Sample names are defined with first digit representing line 6 or 7, second digit representing the biological replicate, and the third character indicating whether the bird was infected with MDV (M) or an uninfected control (C). (**B**,**C**) Principal component analysis of bulk-collated gene expression data, showing PC1 versus PC2 (**B**) and PC1 versus PC3 (**C**). (**D**) Viral TPM values for each cell, grouped by cluster, shows signs of infection in most T cell clusters, as well as the plasma B and antigen-presenting myeloid clusters. (**E**,**F**) Viral TPMs for only cells from challenged birds, broken down by line, demonstrate that more clusters in the susceptible line show signs of infection than in the resistant line.

To investigate which cells showed signs of viral transcription and, thus, evidence of infection with MDV, we aligned the scRNAseq reads to the chicken reference concatenated with the MDV reference genome and counted transcripts aligning to the viral genome in each cell. As expected, the infected samples showed significantly higher levels of viral transcript expression than the uninfected samples (t-test *p* = 0.0072; Fig. 3A). All uninfected birds had total viral transcripts per million (TPM) under 2 (mean = 1.46; STD = 0.225). The viral load of the infected birds was more variable, as has been seen previously^34^ showing four of the six infected birds had a viral TPM higher than 10, but the TPMs of the other two (63 M: TPM = 1.86; 72 M: TPM = 3.01) were closer to those of the uninfected birds than the other infected birds (for all six infected birds, mean = 14.2, STD = 9.62). However, a principal components analysis of gene expression at the sample level shows 72 M clustering with other infected birds despite its low viral TPM, with PC1 corresponding to infection status (Fig. 3B) and PC3 corresponding to line (Fig. 3C). The position of 63 M as an outlier in both PCA and TPM reflects the previously known high variability of the timing of MDV disease progression in chickens^34^. Viral load by transcript analysis generally reflected viral genome copy numbers by quantitative PCR (Suppl. Figure 3), apart from 71 M, which showed a higher viral genome titer in relation to other samples, versus its comparatively lower viral transcript load; this may also be reflective of variation in phase of viral replication.

An examination of viral transcription by cell type and line among the infected birds shows viral transcription in all T cell clusters except effector T cells, as well as plasma B cells and antigen-presenting myeloid cells, but no viral transcription in antigen-presenting B cells, macrophages, or granulocytes (Fig. 3D). In contrast to the susceptible line, for challenged MD resistant birds, viral transcripts were only detectable in the bulk T cell cluster (Fig. 3E–F).

### Cell type abundance changes upon MDV infection within line

**Figure 4:**
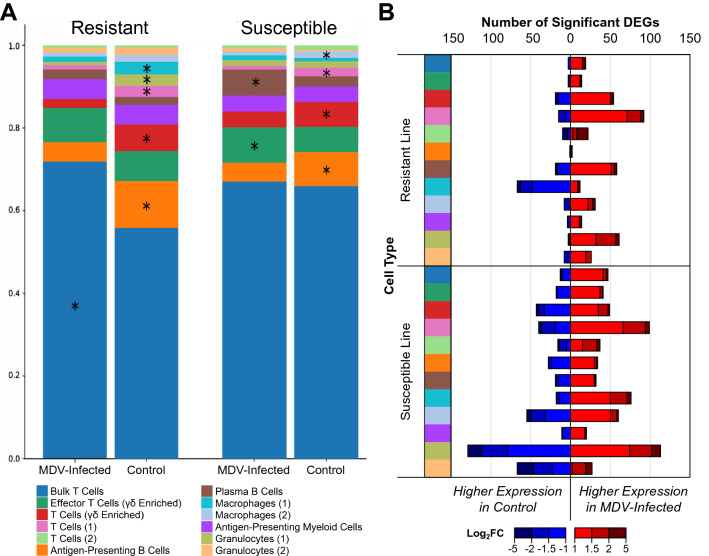
MDV infection shifts cell type composition and gene expression profiles. (**A**) Cell type composition by infection status within the resistant (ADOL 6_3_) or susceptible line (ADOL 7_2_). Each cell type is indicated by color according to the key. Within each line, significant differences (*p* < 0.05) in abundance between the MDV-infected and controls are marked by asterisks on whichever infection status had higher abundance of that cell type. (**B**) Summary of significant differentially expressed genes (DEGs; |log_2_FC|≥ 1.0, *p*-adj < 0.01) in MDV-infected compared to control for each cell type within the resistant and susceptible lines (total n = 964). The number of DEGs increased by MDV are represented by the red bars (right side of the plot), while DEGs decreased by MDV are shown in blue (left side). The magnitude of log_2_FC for the DEGs within each bar is further illustrated by binning into the red-blue color scale.

Upon prediction of putative cell types, we estimated the proportional changes for each cell type using a Fisher’s Exact test (*p* < 0.05) when comparing within susceptible or resistant lines for MDV-infected versus control. Within each line, generally few cell types showed significant compositional shifts upon MDV-infection (Fig. 4A) with only MDVinfected resistant birds showing a significant increase in the bulk T cells (Fig. 4A). In both lines, the proportion of T cells (2) and T cells (γδ enriched) decreased with viral infection. Antigen presenting B cells were lower in the infected state for both lines, while plasma B cells only increased in abundance among susceptible birds. Among myeloid cell types, very few significant changes were observed for cell counts between infected and control within line (Fig. 4A), although granulocytes (1) were lower in the resistant line upon MDV infection.

### General differential gene expression responses to MDV infection within line

**Figure 5:**
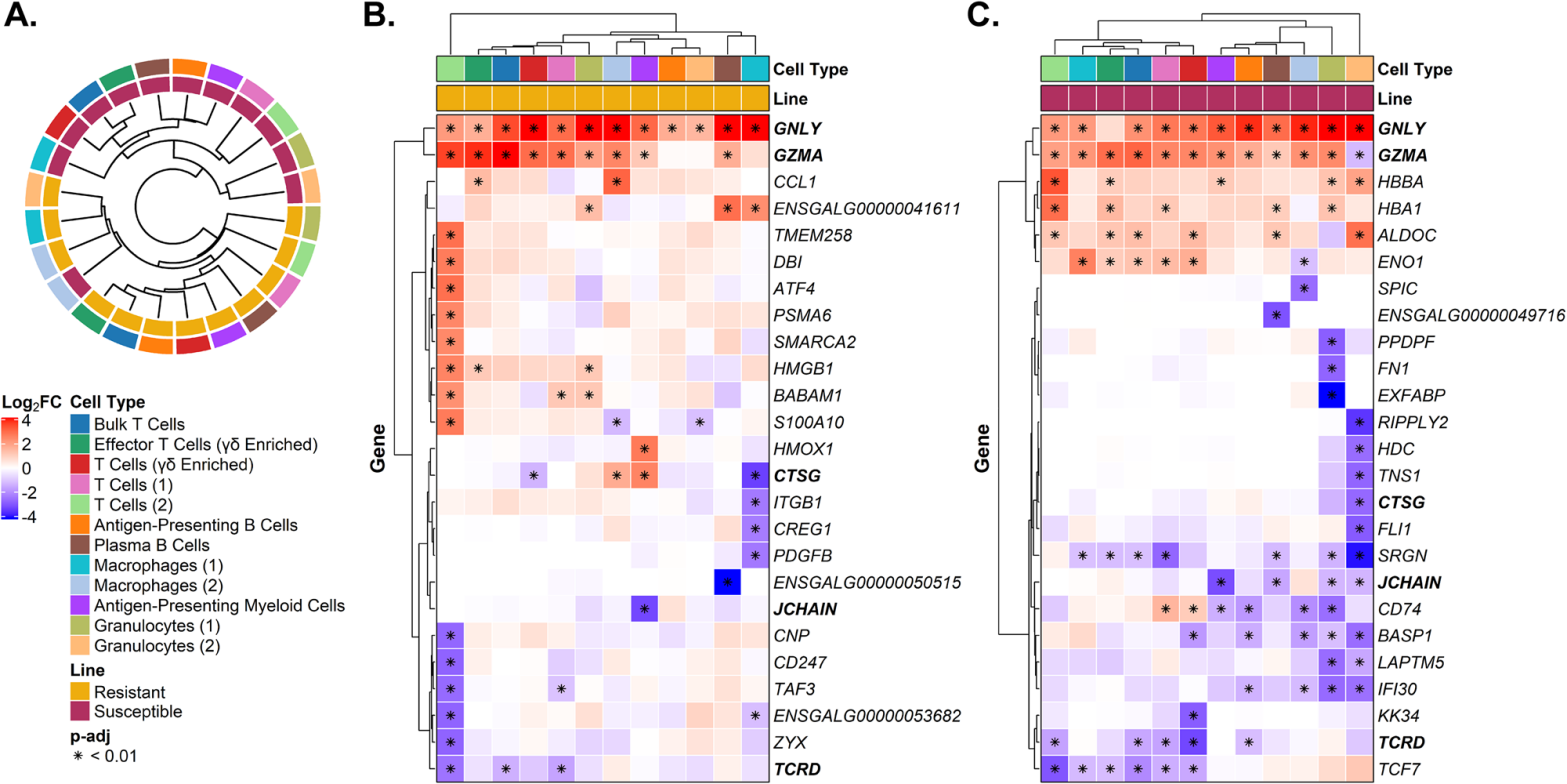
Genetic lines and cell types differ in their response to MDV infection. (**A**) Hierarchical clustering of each cell type in each line based on the log_2_ fold change (log_2_FC; MDV-infected compared to control) of all genes with significant differential expression in at least one contrast (n = 964). (**B**) Log_2_FC across cell types for the top 25 differentially expressed genes (DEGs) in the resistant line (ADOL 6_3_). (**C**) Log_2_FC across cell types for the top 25 DEGs in the susceptible line (ADOL 7_2_). The magnitude of log_2_FC is shown according to the red-blue color scale. Cell type and genetic line are also annotated by color on the dendrogram and heatmaps. Significant log_2_FCs (|log_2_FC|≥ 1.0, *p*-adj < 0.01) are indicated by an asterisk and those genes with significance in both lines are shown in bold.

The total number of DEGs (*p* < 0.01) upon viral infection across all cell types was 1.9-times greater in the MD susceptible birds than in resistant birds (Fig. 4B; Suppl. Tables 2 and 3). Hierarchical clustering revealed that the major separation between cell clusters (except for macrophages (2)) was based on genetic line rather than cell type, consistent with the large differences in resistance to MD between these lines (Fig. 5A). Along with a general feature of higher numbers of DEGs seen in the susceptible birds, several cell type-specific differences were found between lines (Fig. 5B,C). For example, a larger number of down-regulated DEGs was observed in macrophages (1) but only in the resistant line during MDV infection (Figs. 4B, 5B,C). The cell types with the most upregulated genes upon MDV infection in the susceptible line were granulocytes (1) then T cells (1) (Figs. 4B and 5C).

When examining specific genic responses to MDV, granulysin (*GNLY*) and granzyme A (*GZMA*) showed near universal significant changes in expression across cell types in both lines (Fig. 5B,C); however, some cell type variabilities of these cell perforating genes were observed, such as fewer cell types increasing expression of *GZMA* in the resistant line (Fig. 5B,C). More broadly, transcriptomic responses to MDV in certain cell types also revealed the importance of genes with limited prior annotation. For example, *ENSGALG00000043654* (*TCRD*) was previously identified based on aligned mRNA datasets as the TCR δ locus on chromosome 27^35,36^, and in this study was a DEG in three lymphoid cell types of resistant birds and five in susceptible birds (Fig. 5B,C). Despite their importance and significant prior study, the B and T cell antigen receptor loci lack full annotation in the chicken genome. Given the enormity of DEG responses across cell types we highlight mostly immune genes of interest based on our prior studies of MDV in the chicken^9^.

### Bulk T cells

In both lines, *TCF7*, a transcriptional activator in T cell lymphocyte differentiation^37^, was most downregulated in response to MDV. Similarly, in both lines, an additional member of the cytotoxic serine protease gene family, granzyme K (*GZMK*; *ENSGALG00000013546*) was upregulated in MDV-infected compared to control (Suppl. Tables 2 and 3). Overall, very few genes were downregulated (n = 3 or 13; Fig. 4B; Suppl. Tables 2 and 3) in either line when MDV-infected, with a greater upregulation of genes (n = 20 or 48; Fig. 4B; Suppl. Tables 2 and 3) in this T cell population.

### Effector T cells (γδ enriched)

As in the bulk T cell cluster, total DEGs in effector T cells were higher in susceptible (n = 60 DEGs) than resistant birds (n = 18) (Fig. 4B; Suppl. Tables 2 and 3). Chemokine receptor genes (C–C motif chemokine ligand 1 (*CCL1*)*,* C-X3-C chemokine receptor 1 (*CX3CR1*)) expression levels were elevated by MDV infection in this cell type only within resistant birds (Suppl. Tables 2 and 3). Similarly, *BLB2* expression was higher only in the resistant line (Suppl. Tables 2 and 3).

### T cells (γδ enriched)

**Figure 6:**
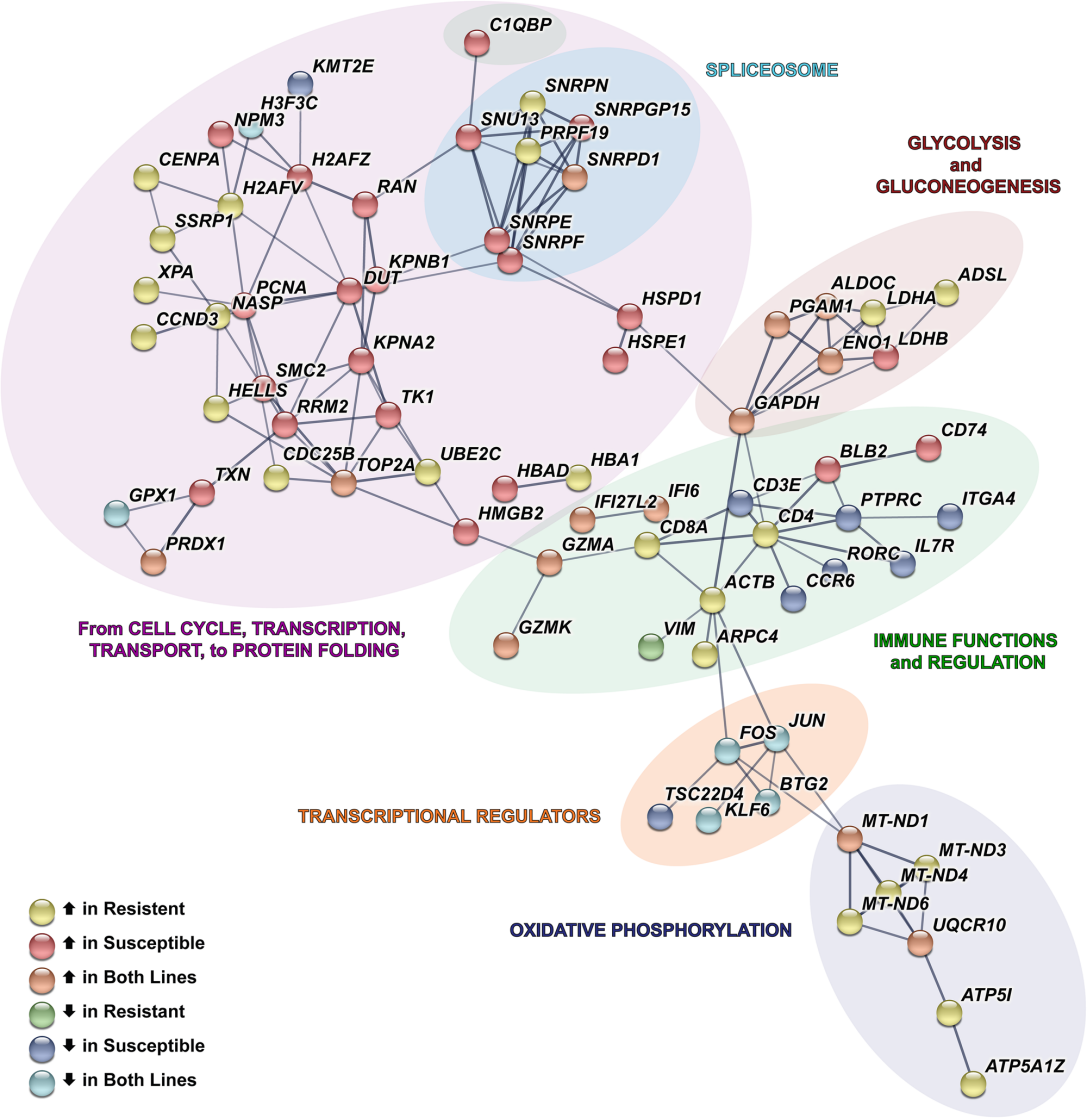
Network of DEGs in T cells (γδ enriched) after MDV infection. Within this cell type, connections between the significant differentially expressed genes (DEGs) from both lines cumulatively were identified using STRING (PPI enrichment *p* =  < 1.0e − 16). For each DEG, the direction of log_2_FC (MDV-infected compared to control) is shown by the fill color for its node. Edges (line thickness) represent the strength of connections (interaction scores) between DEGs. Background colors represent broad-level functions associated with the DEGs in each branch of the network.

Upon MDV infection for splenic T cells (γδ enriched), we identified 93 and 73 DEGs in the susceptible and resistant lines, respectively (Fig. 4B; Suppl. Tables 2 and 3). As in the previous T cell clusters, multiple cytotoxic serine proteases, not just *GZMA*, responded to MDV, with increased *GZMK* and decreased granzyme G-like gene (*ENSGALG00000054174*) expression within both lines (Suppl. Tables 2 and 3). Other immune related genes significantly changed expression within MDV-infected birds, and when searching protein–protein interaction networks using STRING, we find the expected immune functions within these T cells’ (γδ enriched) DEGs as well as more generalized molecular processes such as transcription, glycolysis, and oxidative phosphorylation that may indicate changes in T cell metabolism and activation state (Fig. 6).

### T cells (1 and 2)

These T cells represent undefined types. For resistant line T cells (1), 92 and 15 genes were up- or down-regulated, respectively (Fig. 4B; Suppl. Table 3). In both cell types expression of *IL7R* was significantly decreased (Supp. Tables 2 and 3). Upon infection in T cells (2), the total number of DEGs was larger in susceptible compared to resistant birds, 53 versus 32, respectively (Suppl. Tables 2 and 3; Fig. 4B). In susceptible birds, a significant decrease in *TCF7* gene expression occurred in T cells (2) upon MDV infection; we observed this same decrease in both lines for the bulk T cells and effector T cells (γδ enriched), and specifically in the susceptible line in T cells (γδ enriched) and T cells (1) (Suppl. Tables 2 and 3).

### B cells

In antigen-presenting B cells, the resistant line had a paucity of DEGs (n = 3) in contrast to susceptible birds (n = 62 DEGs) (Fig. 4A,B). For susceptible birds, *IFI6* increased while *IGLL1*, the invariant (class II) chain CD74 molecule (*CD74*), and *CD79B* decreased during MDV infection (Suppl. Table 2). In plasma B cells, a higher number of DEGs was seen in the resistant line, 77 compared to 51 in susceptible birds with some down regulation events of immune interest in resistant birds such as interleukin 1 receptor associated kinase 2 (*IRAK2*; Suppl. Table 3).

### Macrophages (1 and 2)

**Figure 7:**
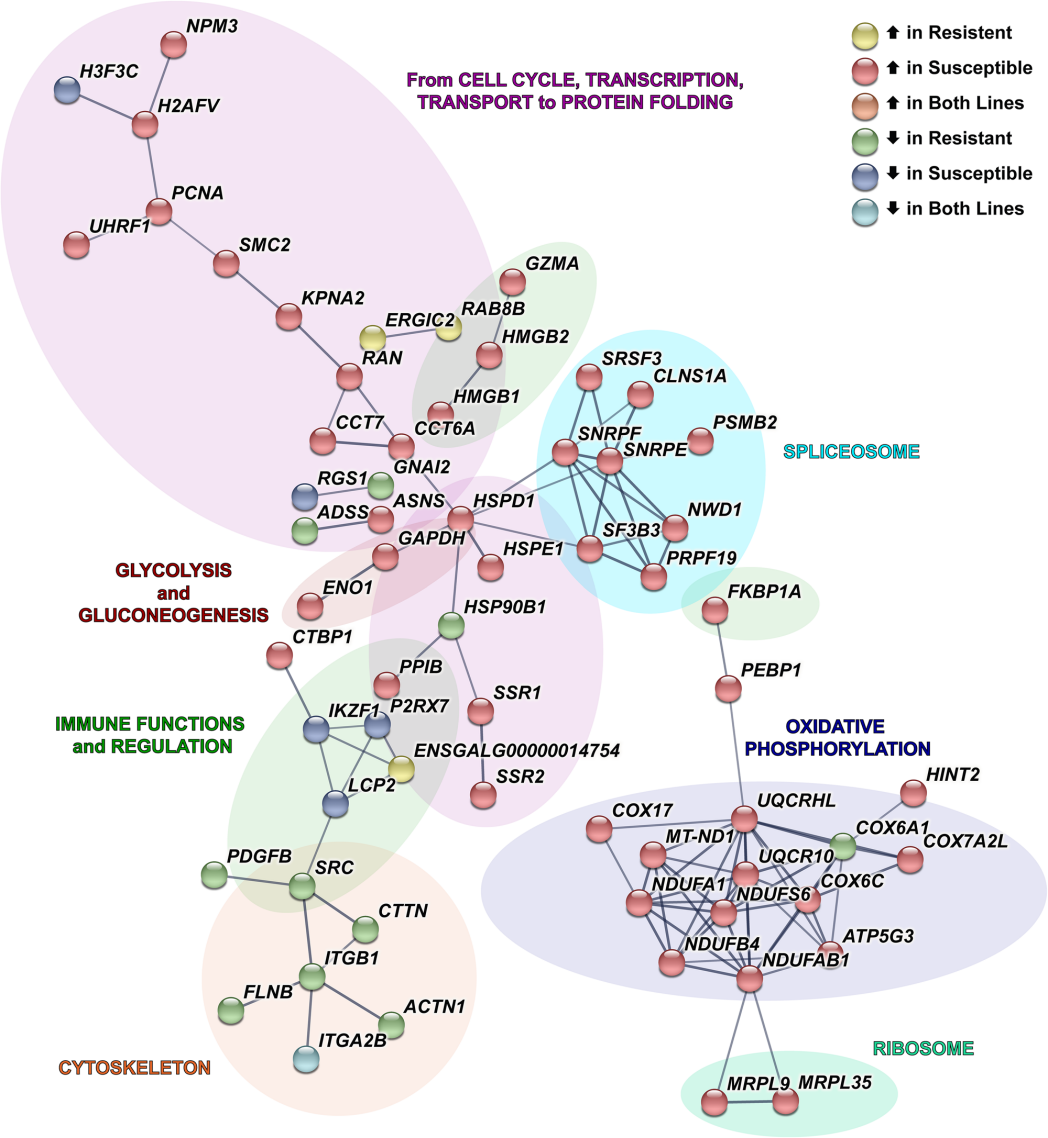
Network of DEGs in macrophages (1) after MDV infection. Within this cell type, connections between the significant differentially expressed genes (DEGs) from both lines cumulatively were identified using STRING (PPI enrichment *p* = 1.19e − 14). For each DEG, the direction of log_2_FC (MDV-infected compared to control) is shown by the fill color for its node. Edges (line thickness) represent the strength of connections (interaction scores) between DEGs. Background colors represent broad-level functions associated with the DEGs in each branch of the network.

Transcriptional responses to MDV infection of susceptible and resistant birds within macrophages (1) showed 94 and 79 total DEGs, respectively (Suppl. Tables 2 and 3). Within the resistant line, only 15% of DEGs were upregulated with the inverse seen in the susceptible line, 81%. The highest upregulated DEG for susceptible macrophages (1) was enolase 1 (*ENO1*), an emerging gene involved in cell transformation^38^ (Suppl. Table 2). The DEG number within macrophages (2) of resistant birds was much lower (n = 39 genes) than susceptible birds (n = 116 genes; Suppl. Tables 2 and 3). Protein–protein interactions were predicted, and for macrophages (1), revealed a much larger number of connections due to susceptible DEGs, especially those involved in transcription, splicing, and oxidative phosphorylation (Fig. 7). Most DEGs, irrespective of macrophage type and resistant or susceptible line, were unique.

### Antigen-presenting myeloid cells

These cells showed a moderate transcriptional response in both lines, with 31 and 18 DEGs in susceptible and resistant (Suppl. Tables 2 and 3). In resistant birds, two genes affiliated with the interferon activation pathway were increased upon infection, TNF alpha induced protein 2 (*TNFAIP2*) and interferon induced protein with tetratricopeptide repeats 5 (*IFIT5*). In contrast, *JCHAIN* is downregulated in both lines during viral infection (Suppl. Tables 2 and 3). Class II antigen presentation is decreased as seen with lower *BLB2* and *CD74* gene expression in only susceptible birds (Suppl. Table 2).

### Granulocytes (1 and 2)

Among all cell types, transcriptional responses to infection were the most pronounced (n = 306) in granulocytes (1). Four-fold more DEGs were observed in the susceptible (n = 242) than in the resistant lines (n = 64; Fig. 4B; Suppl. Tables 2 and 3). GRB10 interacting GYF protein 2 (*GIGYF2*), a gene putatively involved in regulating tyrosine kinase receptor activity and *RAP1B*, a member of the RAS oncogene family, are both upregulated in the resistant line, highlighting the diversity of virally-induced responses in this myeloid cell cluster (Suppl. Table 3). In granulocytes (2), total DEGs in susceptible and resistant lines were more modest at 94 and 34, respectively (Suppl. Tables 2 and 3). Among MDV-infected resistant birds, the largest increase in expression was for the 2’-5’ oligoadenylate synthetase gene (*OASL*), known to be indirectly associated with the interferon gamma signaling pathway and antiviral activity^39^ (Suppl. Table 3) while decreased expression of the tumor protein D52-like 2 (*TPD52L2*) gene may suggest some involvement in blunting the tumorigenic properties of MDV^40^.

## Discussion

MDV remains enigmatic, from its ability to continually evolve and evade vaccine protection to the multiplicity of presentations such as tumor formation, and as a result causes substantial economic losses to the poultry industry. This avian single cell study of host transcriptional response to a virus establishes initial criteria for identifying cell types in the chicken spleen, estimating resulting changes in cell abundance and gene expression by cell type, and presents new molecular networks to study the resistance phenotype to an avian oncogenic herpesvirus. Several viral infection studies demonstrate the immense complexity of the immune response^41–44^, which is supported by our chicken scRNAseq data. Despite generating a multitude of testable hypotheses, we focused on those genes or their networks that are likely the most critical to MD resistance.

Despite the limitations faced in the detection of expressed genes with the general use of single cell transcriptomic methods, we find robust gene expression responses to MDV in this experiment*.* When considering all DEGs in both lines, more genes are upregulated during this stage of infection in MD susceptible than resistant birds, as seen previously^9^. We hypothesize that some of this difference is due to more cells in susceptible birds being infected early^9^ but surprisingly two genes, *GZMA* and *GNLY,* both associated with apoptosis events in the virally infected cell, dominate innate and adaptive cell lineages responses across lines. Accumulating evidence suggests a dual role for granzymes that extends beyond just killing infected cells but also protection against viral infection in noncytotoxic ways^45^ and, although most notably expressed in cytotoxic T and NK cell lineages, can be induced in other lymphocyte and non-lymphocyte cell types^46–49^. Our results suggest their functional role could be an aggressive first step of the immune system but perhaps less cell-type specific in MD. *GZMA* is upregulated in response to avian leukosis virus in chicken peripheral blood leukocytes and was suggested to be an important mechanism for cell-mediated cytotoxicity for initial control of viral spread^50^. Sarson et al.^51^ reported that *GZMA* expression increased in splenocytes assayed with an immune-specific microarray after MDV infection in both resistant and susceptible chicken lines, a finding we recapitulate, now with cell type granularity. Vaccination for MD has also been shown to increase *GZMA* and *GNLY* expression in chicken splenic γδ T cells at 3 and 7 dpi^52^ as well as other immunostimulatory gene changes such as increased interferon gamma (*IFNG*)^52,53^. Given the γδ T cells’ importance in human anti-tumor responses^54^, we speculate chickens use this cell type to quickly induce cell perforating responses along with an array of other immune system genes when confronted with MDV.

Chickens have a higher proportion of circulating γδ T cells than many mammalian species^55^, and relevant to this study, splenic γδ T cells are effective mediators of MHC-unrestricted cytotoxicity in chickens^56^. However, their specific role among T cell types in the context of MDV infection has been missing^55^. When T cells (γδ enriched) DEGs in both lines were searched against a protein–protein interactions database multiple networks associated with immune cell development, T cell selection, transcriptional regulation, metabolic responses, and mitochondrial energy production were discovered but, interestingly, some within-network gene regulation is specific to a line (see Fig. 6). Further measurements of their relative abundances at multiple timepoints will be required to estimate their full contribution to MD resistance.

A premise in the adaptive B cells defense against viral pathogens is the generation of high-affinity antibodies requiring longer time periods to contribute to MD resistance. Nonetheless, we show transcriptomic activation differences by line in both B cell types putatively identified in the spleen. For antigen-presenting B cells, very few DEGs are observed upon viral infection in the resistant versus the susceptible line. In contrast, plasma B cells had higher numbers of DEGs in the resistant line upon viral infection, with some genes garnering interest for their potential contribution to resistance, e.g., *IRAK2*, a key component of the toll like receptor complex activation, which can be subdued by vaccinia virus protein interactions^57^. Our data suggest experiments to explore MDV interaction with *IRAK2* could be informative but also a need to look beyond splenic B cell types, e.g., to bursa of Fabricius.

Cells of myeloid origin are additional key players specific to the innate response to early viral infection. In the mixedpopulation of antigen-presenting myeloid cells, two subtypes of granulocytes and macrophages were present. In the antigen-presenting myeloid cells, DEGs overall suggest heightened antigen processing activity. Previously an MDV-protein interaction screen identified proteins that overlap with these DEGs in both lines such as MHC class II beta (*BLB*), *CD74*, and complement component Clq-binding protein (*C1QBP*)^58^. But we also find DEGs in our myleloid cell types with unknown association to MD such as placenta-specific gene 8-like 2 (*PLAC8*). Of note, *PLAC8* research suggests a multifaceted role in tumorigenesis^59^, but its contribution in MD is unknown. Macrophages represent another fundamental means to diminish viral infection^12,60^ and a RNAseq analysis of in vitro MDV-infected macrophages showed significant gene expression changes in cells from both resistant and susceptible birds^12^. Within this study, the role of macrophages in MD resistance is better revealed with changes depending on line and subtype. When comparing these in vitro results to the macrophage clusters identified in this experiment, more DEGs were shared in cells/clusters from susceptible birds (29 genes), including for example, increased *CCL1* and decreased *MARCO* in the macrophage (2) cluster, than cells/clusters from resistant birds (8 genes). The biological meaning of this in vivo transcriptomic shift toward a susceptible or resistant phenotype merits further experimentation with macrophage subtypes.

Allele specific expression (ASE) in MDV host response^9^ and genes embedded within QTLs^10^ previously associated with MDV responses offered us opportunities to prioritize gene candidates among our DEGs by cell type. A previous MDV-challenge study conducted at 4 dpi, identified 20 higher-priority QTL candidate genes based on various factors^10^. Two candidates match our DEGs. Plasma B cells of the resistant line that expressed the ADAM metallopeptidase domain 10 (*ADAM10*)), which is from the same gene family, yet had unknown functional similarity to the QTL candidate ADAM metallopeptidase thrombospondin type 1 motif 5 (*ADAMTS5*)^10^ in chicken; the second QTL candidate gene, *CD79B,* was down-regulated only in antigen-presenting B cells from MDV-infected susceptible birds (Suppl. Table 2). Despite few QTL-associated genes overlapping MD DEGs in this study, as a collated candidate gene set, we expect modules of larger gene regulatory networks could be tested for trait association in the future. Moreover, many other non-immune genes with possible viral protection roles to play such as the tumor suppressor RAS and EF-hand domain containing (*RASEF*) gene*,* expressed only during the resistant line response, should be considered.

Unlike prior MDV challenge studies^9,12^, we find novel cell type specific responses that when taken together provide more insight into the various immune system components at play. Our results show distinct gene expression differences, for instance, when in vivo splenic macrophages are exposed to MDV compared to in vitro infection^12^. The molecular mechanisms that underlie the multifaceted phenotypes of MDV response suggest many canonical signaling pathways are affected with varying temporal elements to each, therefore leaving much to explore and validate with this data set in future experiments.

## Methods

### Experimental design

Ten birds each from the MD-susceptible (ADOL 7_2_) and -resistant (ADOL 6_3_) lines were placed into Horsfall-Bauer (HB) units at hatch, five birds per line and HB unit. At one week of age, five birds of each line (1 HB unit) were challenged intra-abdominally with 2,000 pfu MDV (JM/102W strain). Due to the need to process multiple samples and quickly transport them to an offsite facility, uninfected control birds and MDV-infected birds were obtained three weeks apart from separate hatches, but all birds were age-matched at euthanasia to six days after MDV infection. For these experiments, we used CO_2_ gas euthanasia, following the current standards for poultry euthanasia provided in AVMA Guidelines for the Euthanasia of Animals (2020 Edition). All experiments presented herein were carried out in accordance with the approval of the Institutional Animal Care and Use Committee, USDA, ARS, ADOL, East Lansing, MI (protocol approval number 2018–01). Moreover, all methods were performed in accordance with the ARRIVE guidelines.

### Library preparation and sequencing

After euthanizing the birds, spleens were aseptically removed, homogenized, filtered to single-cell suspension through 35 micron cell strainers (Fisher Scientific, Waltham, MA), enriched for mononuclear leukocytes over Histopaque-1077 (Millipore Sigma, Burlington, MA), and transferred the same day on ice for single-cell capture and library preparation of a targeted 3,000 cells/sample. Single cell capture and cDNA library preparation were performed on the 10 × Genomics Chromium Single Cell 3’ instrument (10 × Genomics, Pleasanton, CA) according to the manufacturer’s recommendations. Pooled libraries were barcoded by sample and sequenced (2 × 150 bp length) on an Illumina HiSeq 4000 (Illumina, San Diego, CA).

### Read alignment and processing

The individual tissue-specific sequenced Gel Bead-In Emulsion (GEM) libraries were each initially processed with the Cell Ranger (v3.1.0) pipeline (10 × Genomics), which performs demultiplexing, alignment, barcode processing, and sample aggregation to create a cellular barcode by genomic feature matrix, as described^61^. The GRCg6a genome reference (GCA_000002315.5) was used for all sequence alignments, and the accompanying Ensembl gene coordinate files were used to facilitate gene identification.

### Filtering, normalization, and clustering

We used the scanpy platform v1.5.2^62^ to filter and normalize the cell by feature counts matrix, and to perform all subsequent analysis. First, we loaded the aggregated filtered feature matrix from the Cell Ranger output and filtered out cells expressing fewer than 200 or more than 1,000 genes, cells with a total UMI count of more than 2,500, and cells with more than 20% of counts mapping to mitochondrial genes or more than 50% of counts in ribosomal protein genes. We normalized counts per cell, logarithmized the resulting matrix, and scaled genes to unit variance and zero mean, and regressed out total counts per cell and mitochondrial and ribosomal count percentages. We calculated principal components using only highly variable genes, selected by the “highly_variable_genes” function in scanpy and batch-corrected the results by sample using Harmony^18^. We computed the 10-nearest-neighbor graph with the first 20 corrected principal components, and then used the Leiden algorithm^20^ with resolution parameter set to 0.3 to partition the graph into clusters. We used uniform manifold and approximation projection (UMAP) dimensionality reduction^19^ to visualize the results. A Jupyter notebook containing all code used to perform these steps is available in this project’s software repository and is available upon request.

### Cell type identification and proportional change

We computed marker genes for each cluster using scanpy’s default t-test with overestimated variance. Specific gene biomarkers are not yet known for most *Gallus gallus* cell types we expected to identify in the sampled tissue, the spleen; we therefore used a manual curation approach, starting with known avian cell type-specific gene markers from the literature (e.g., *CD3E* for T cells), and then when necessary indirect inference from orthologous known human or mouse gene-specific cell type markers that are cataloged in CellMarker^63^ and PanglaoDB^64^. In some cases we used previously identified immune cell types in human and mouse spleen^65,66^. For each cluster, we ranked the top 20 differentially expressed genes (DEGs) (*p* < 0.01 value) when compared to all other clusters, then used these DEGs to assign cell type identity to each cluster. At this first stage of clustering, one cluster of cells overwhelmingly represented by genes involved in cell cycle progression was removed and clustering was repeated on the remaining cells using identical parameters except for a Leiden resolution of 0.25. The final top 20 DEGs were again used to finalize our cell type identities as described above (Supp. Table 1).

Once our final clusters were labelled by cell type, we calculated the proportion of cell types, aggregating by line and treatment groups. To determine whether a given cell type was proportionally over- or under-represented after infection compared to control in the susceptible or resistant line, we used a Fisher’s exact test.

### Viral transcription

We calculated viral transcript counts by taking the sum of counts per cell for all UMIs mapping to the Marek's disease virus genome (Genbank ID: AF147806.2). We normalized these counts within each individual using the transcripts per million method.

### Viral titers by quantitative PCR

We quantitated viral genomes/cell equivalent in stored frozen aliquots of the Histopaque-1077-enriched splenocyte samples using previously published methods^67,68^. In brief, total DNA was extracted and purified over silica columns using Qiagen DNAEasy reagents (Qiagen, Germantown, MD). qPCR was performed using the Taqman Fast Universal PCR kit (Thermo Fisher Scientific, Waltham, MA) with primers that amplify chicken GAPDH and MDV glycoprotein B. Standard curves for absolute quantitation were prepared from plasmids containing chicken GAPDH or MDV gB genes, in tenfold serial dilution. A total of 40 amplification cycles were performed on a BioRad CFX96 Real-Time system (BioRad Laboratories, Hercules, CA), and viral genomes / cell equivalent was calculated as (copies gB / copies GAPDH) * 2 GAPDH copies/cell.

### Bulk principal components analysis

To perform principal components analysis at a sample level instead of a cell level, we took the sum of all counts for cells in each library to convert the cell by gene matrix to a sample by gene matrix and normalized using the transcripts per million method. We then performed principal component analysis on the resulting matrix.

### Differential gene expression

We used edgeR^69^ with the QLF test and cellular detection rate as a covariate to test for the differential expression of genes by cell type between pairs of groups of birds as described in Soneson and Robinson (2018)^70^, which showed this method to be robust and scalable in the number of genes detected with the fewest false positives. We performed the following two pairwise comparisons for each identified cell type: infected versus control within resistant or within susceptible lines. In this study any gene with a log_2_FC > 1.0 and *p* < 0.01 was defined as differentially expressed. We found the *HINTW* gene to show differential expression in some instances due to its multicopy presentation on the W chromosome, so it was removed from further consideration after completing our statistical tests. The DEGs were also investigated by overlap with known protein–protein interactive networks using STRING^71^ and the most informative networks are shown (see Figs. 6 and 7).

## Supplementary Information

Supplementary Table 1.

Supplementary Table 2.

Supplementary Table 3.

Supplementary Figures.

## Data Availability

The datasets generated during and/or analyzed during the current study are available in the Gene Expression Omnibus repository under accession GSE202739.

## References

[1] Bacon LD, Hunt HD, Cheng HH (2000). A review of the development of chicken lines to resolve genes determining resistance to diseases. Poult. Sci..

[2] Dunn JR, Black Pyrkosz A, Steep A, Cheng HH (2019). Identification of Marek's disease virus genes associated with virulence of US strains. J. Gen. Virol..

[3] Hunt HD (2001). Marek's disease virus down-regulates surface expression of MHC (B Complex) Class I (BF) glycoproteins during active but not latent infection of chicken cells. Virology.

[4] Sun GR (2019). Differential expression of type I interferon mRNA and protein levels induced by virulent Marek's disease virus infection in chickens. Vet. Immunol. Immunopathol..

[5] Li K (2019). Avian oncogenic herpesvirus antagonizes the cGAS-STING DNA-sensing pathway to mediate immune evasion. PLoS Pathog..

[6] Bertzbach LD (2019). The transcriptional landscape of Marek's disease virus in primary Chicken B cells reveals novel splice variants and genes. Viruses.

[7] Kennedy DA (2017). Industry-wide surveillance of Marek's disease virus on commercial poultry farms. Avian Dis..

[8] Schat KA, Xing Z (2000). Specific and nonspecific immune responses to Marek's disease virus. Dev. Comp. Immunol..

[9] Cheng HH (2015). Fine mapping of QTL and genomic prediction using allele-specific expression SNPs demonstrates that the complex trait of genetic resistance to Marek's disease is predominantly determined by transcriptional regulation. BMC Genom..

[10] Smith J, Lipkin E, Soller M, Fulton JE, Burt DW (2020). Mapping QTL associated with resistance to Avian Oncogenic Marek's disease virus (MDV) reveals major candidate genes and variants. Genes (Basel).

[11] Kaya M, Preeyanon L, Dodgson JB, Cheng HH (2016). Validation of alternative transcript splicing in chicken lines that differ in genetic resistance to Marek's disease. Anim. Biotechnol..

[12] Chakraborty P (2019). Macrophages from susceptible and resistant chicken lines have different transcriptomes following Marek's disease virus infection. Genes (Basel).

[13] Trapp-Fragnet L (2021). Marek's disease virus prolongs survival of primary chicken B-cells by inducing a senescence-like phenotype. PLoS Pathog..

[14] Speranza E (2021). Single-cell RNA sequencing reveals SARS-CoV-2 infection dynamics in lungs of African green monkeys. Sci. Transl. Med..

[15] Steuerman Y (2018). Dissection of influenza infection in vivo by single-cell RNA sequencing. Cell. Syst..

[16] Shah AU (2021). From nasal to basal: single-cell sequencing of the bursa of Fabricius highlights the IBDV infection mechanism in chickens. Cell Biosci..

[17] Qu X, Li X, Li Z, Liao M, Dai M (2022). Chicken peripheral blood mononuclear cells response to Avian Leukosis virus subgroup J infection assessed by single-cell RNA sequencing. Front. Microbiol..

[18] Korsunsky I (2019). Fast, sensitive and accurate integration of single-cell data with Harmony. Nat. Methods.

[19] Becht E (2018). Dimensionality reduction for visualizing single-cell data using UMAP. Nat. Biotechnol..

[20] Traag VA, Waltman L, van Eck NJ (2019). From Louvain to Leiden: Guaranteeing well-connected communities. Sci. Rep..

[21] Houssaint E, Lassila O, Vainio O (1989). Bu-1 antigen expression as a marker for B cell precursors in chicken embryos. Eur. J. Immunol..

[22] Ordway D (2007). XCL1 (lymphotactin) chemokine produced by activated CD8 T cells during the chronic stage of infection with Mycobacterium tuberculosis negatively affects production of IFN-gamma by CD4 T cells and participates in granuloma stability. J. Leukoc. Biol..

[23] Capone A, Volpe E (2020). Transcriptional regulators of T helper 17 cell differentiation in health and autoimmune diseases. Front. Immunol..

[24] Wen Z, Xu L, Xu W, Xiong S (2021). Retinoic acid receptor-related orphan nuclear receptor gammat licenses the differentiation and function of a unique subset of follicular helper T cells in response to immunogenic self-DNA in systemic lupus erythematosus. Arthritis Rheumatol..

[25] Parker A, Kaufman J (2017). What chickens might tell us about the MHC class II system. Curr. Opin. Immunol..

[26] Matulova M (2012). Characterization of chicken spleen transcriptome after infection with Salmonella enterica serovar Enteritidis. PLoS ONE.

[27] Sekelova Z (2017). Differential protein expression in chicken macrophages and heterophils in vivo following infection with Salmonella Enteritidis. Vet. Res..

[28] Thul PJ, Lindskog C (2018). The human protein atlas: A spatial map of the human proteome. Protein Sci..

[29] Sasaki H, Kurotaki D, Tamura T (2016). Regulation of basophil and mast cell development by transcription factors. Allergol. Int..

[30] Lacoste-Eleaume AS (1994). Biochemical and functional characterization of an avian homolog of the integrin GPIIb-IIIa present on chicken thrombocytes. Exp. Cell Res..

[31] Ferdous F (2016). Transcriptome profile of the chicken thrombocyte: New implications as an advanced immune effector cell. PLoS ONE.

[32] Haertle S (2017). Identification of the receptor and cellular ortholog of the Marek's Disease Virus (MDV) CXC chemokine. Front. Microbiol..

[33] Sutton KMM, Borowska D, Sang H, Kaiser P, Balic A, Vervelde L (2021). Characterization of conventional dendritic cells and macrophages in the spleen using the CSF1R-Reporter transgenic chickens. Front. Immunol..

[34] Islam AF, Walkden-Brown SW, Islam A, Underwood GJ, Groves PJ (2006). Relationship between Marek's disease virus load in peripheral blood lymphocytes at various stages of infection and clinical Marek's disease in broiler chickens. Avian Pathol..

[35] Kubota T (1999). Characterization of an avian (Gallus gallus domesticus) TCR alpha delta gene locus. J. Immunol..

[36] Parra ZE, Mitchell K, Dalloul RA, Miller RD (2012). A second TCRdelta locus in Galliformes uses antibody-like V domains: Insight into the evolution of TCRdelta and TCRmu genes in tetrapods. J. Immunol..

[37] Zhang J, Lyu T, Cao Y, Feng H (2021). Role of TCF-1 in differentiation, exhaustion, and memory of CD8(+) T cells: A review. FASEB J..

[38] Chen JM (2020). Enolase 1 differentially contributes to cell transformation in lung cancer but not in esophageal cancer. Oncol. Lett..

[39] Del Vesco AP, Jang HJ, Monson MS, Lamont SJ (2021). Role of the chicken oligoadenylate synthase-like gene during in vitro Newcastle disease virus infection. Poult. Sci..

[40] Zhong A, Chen T, Zhou T, Zhang Z, Shi M (2021). TPD52L2 is a prognostic biomarker and correlated with immune infiltration in lung adenocarcinoma. Front. Pharmacol..

[41] Goodrum F, McWeeney S (2018). A single-cell approach to the elusive latent human cytomegalovirus transcriptome. MBio.

[42] Kotliar D (2020). Single-cell profiling of ebola virus disease in vivo reveals viral and host dynamics. Cell.

[43] Wilk AJ (2020). A single-cell atlas of the peripheral immune response in patients with severe COVID-19. Nat. Med..

[44] Kazer SW (2020). Integrated single-cell analysis of multicellular immune dynamics during hyperacute HIV-1 infection. Nat. Med..

[45] de Jong LC, Crnko S, Ten Broeke T, Bovenschen N (2021). Noncytotoxic functions of killer cell granzymes in viral infections. PLoS Pathog..

[46] Strik MC (2007). Human mast cells produce and release the cytotoxic lymphocyte associated protease granzyme B upon activation. Mol. Immunol..

[47] Kim WJ, Kim H, Suk K, Lee WH (2007). Macrophages express granzyme B in the lesion areas of atherosclerosis and rheumatoid arthritis. Immunol. Lett..

[48] Hagn M, Jahrsdorfer B (2012). Why do human B cells secrete granzyme B? Insights into a novel B-cell differentiation pathway. Oncoimmunology.

[49] Turner CT (2019). Granzyme K expressed by classically activated macrophages contributes to inflammation and impaired remodeling. J. Investig. Dermatol..

[50] Dai M (2020). Systematic Identification of host immune key factors influencing viral infection in PBL of ALV-J infected SPF chicken. Viruses.

[51] Sarson AJ, Parvizi P, Lepp D, Quinton M, Sharif S (2008). Transcriptional analysis of host responses to Marek's disease virus infection in genetically resistant and susceptible chickens. Anim. Genet..

[52] Hao X (2021). An anti-tumor vaccine against Marek’s disease virus induces differential activation and memory response of gammadelta T cells and CD8 T cells in chickens. Front. Immunol..

[53] Laursen AMS (2018). Characterizaton of gamma delta T cells in Marek's disease virus (Gallid herpesvirus 2) infection of chickens. Virology.

[54] Lawand M, Dechanet-Merville J, Dieu-Nosjean MC (2017). Key features of gamma-delta T-Cell subsets in human diseases and their immunotherapeutic implications. Front. Immunol..

[55] Yang Y, Dong M, Hao X, Qin A, Shang S (2020). Revisiting cellular immune response to oncogenic Marek's disease virus: The rising of avian T-cell immunity. Cell Mol. Life Sci..

[56] Fenzl L, Gobel TW, Neulen ML (2017). gammadelta T cells represent a major spontaneously cytotoxic cell population in the chicken. Dev. Comp. Immunol..

[57] Harte MT (2003). The poxvirus protein A52R targets Toll-like receptor signaling complexes to suppress host defense. J. Exp. Med..

[58] Niikura M, Liu HC, Dodgson JB, Cheng HH (2004). A comprehensive screen for chicken proteins that interact with proteins unique to virulent strains of Marek's disease virus. Poult. Sci..

[59] Mao M (2021). Multifaced roles of PLAC8 in cancer. Biomark. Res..

[60] Wang D, Sun S, Heidari M (2018). Marek's disease vaccine activates chicken macrophages. J. Vet. Sci..

[61] Zheng GX (2017). Massively parallel digital transcriptional profiling of single cells. Nat. Commun..

[62] Wolf FA, Angerer P, Theis FJ (2018). SCANPY: Large-scale single-cell gene expression data analysis. Genome Biol..

[63] Zhang X (2019). Cell Marker: A manually curated resource of cell markers in human and mouse. Nucleic Acids Res..

[64] Franzen O, Gan LM, Bjorkegren JLM (2019). PanglaoDB: A web server for exploration of mouse and human single-cell RNA sequencing data. Database (Oxford).

[65] Madissoon E (2019). scRNA-seq assessment of the human lung, spleen, and esophagus tissue stability after cold preservation. Genome Biol..

[66] Kimmel JC (2019). Murine single-cell RNA-seq reveals cell-identity- and tissue-specific trajectories of aging. Genome Res..

[67] Dunn JR, Silva RF (2012). Ability of MEQ-deleted MDV vaccine candidates to adversely affect lymphoid organs and chicken weight gain. Avian Dis..

[68] Steep A (2022). Identification and validation of Ikaros (IKZF1) as a cancer driver gene for Marek's disease virus-induced lymphomas. Microorganisms.

[69] Robinson MD, McCarthy DJ, Smyth GK (2010). edgeR: a Bioconductor package for differential expression analysis of digital gene expression data. Bioinformatics.

[70] Soneson C, Robinson MD (2018). Bias, robustness and scalability in single-cell differential expression analysis. Nat. Methods.

[71] Jensen LJ (2009). STRING 8—A global view on proteins and their functional interactions in 630 organisms. Nucleic Acids Res..

