# Supplementary material for: The immune cell landscape and response of Marek’s disease resistant and susceptible chickens infected with Marek’s disease virus
Source: Sci Rep. 2023 Apr 1;13:5355. doi: 10.1038/s41598-023-32308-x (PMC10067856; doi:10.1038/s41598-023-32308-x)
Supplement: Supplementary file 4 — Supplementary Figures. [file 41598_2023_32308_MOESM4_ESM.pdf]

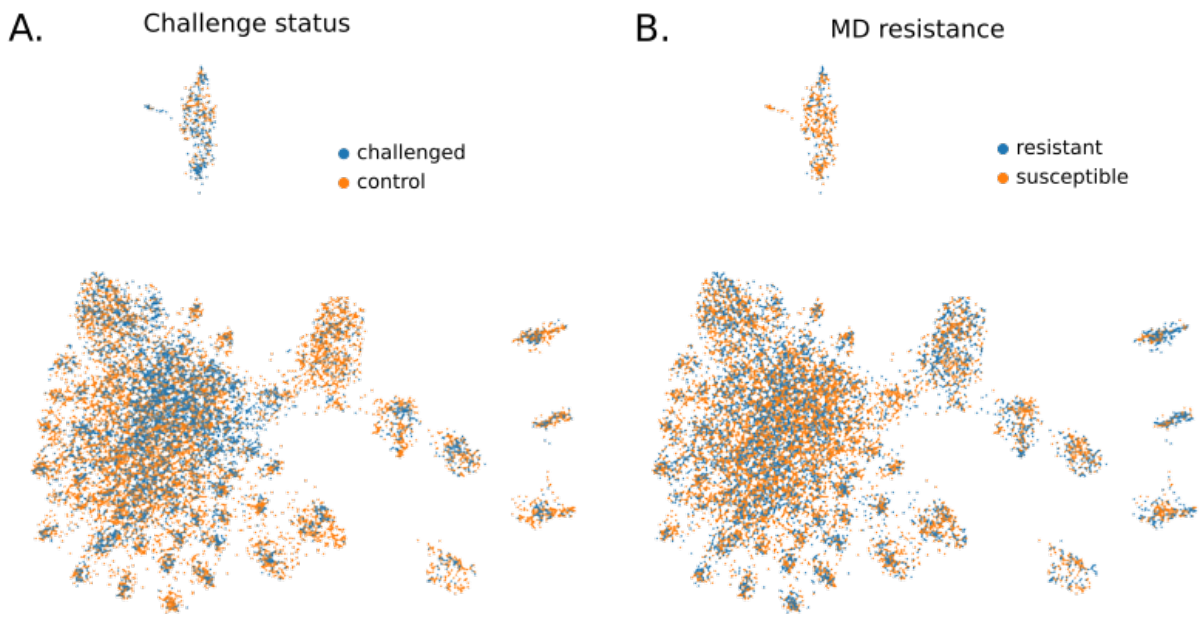

Supplement Figure 1. The integration of cells by cluster across samples for MDV challenge or genetic line. A correction was performed within the principal components space to iteratively remove batch effects. A. All cells associated with individual clusters were parsed by MDV challenge or control assignment and colorized accordingly. B. All cells associated with individual clusters were parsed by the resistant or susceptible line status and colorized accordingly.

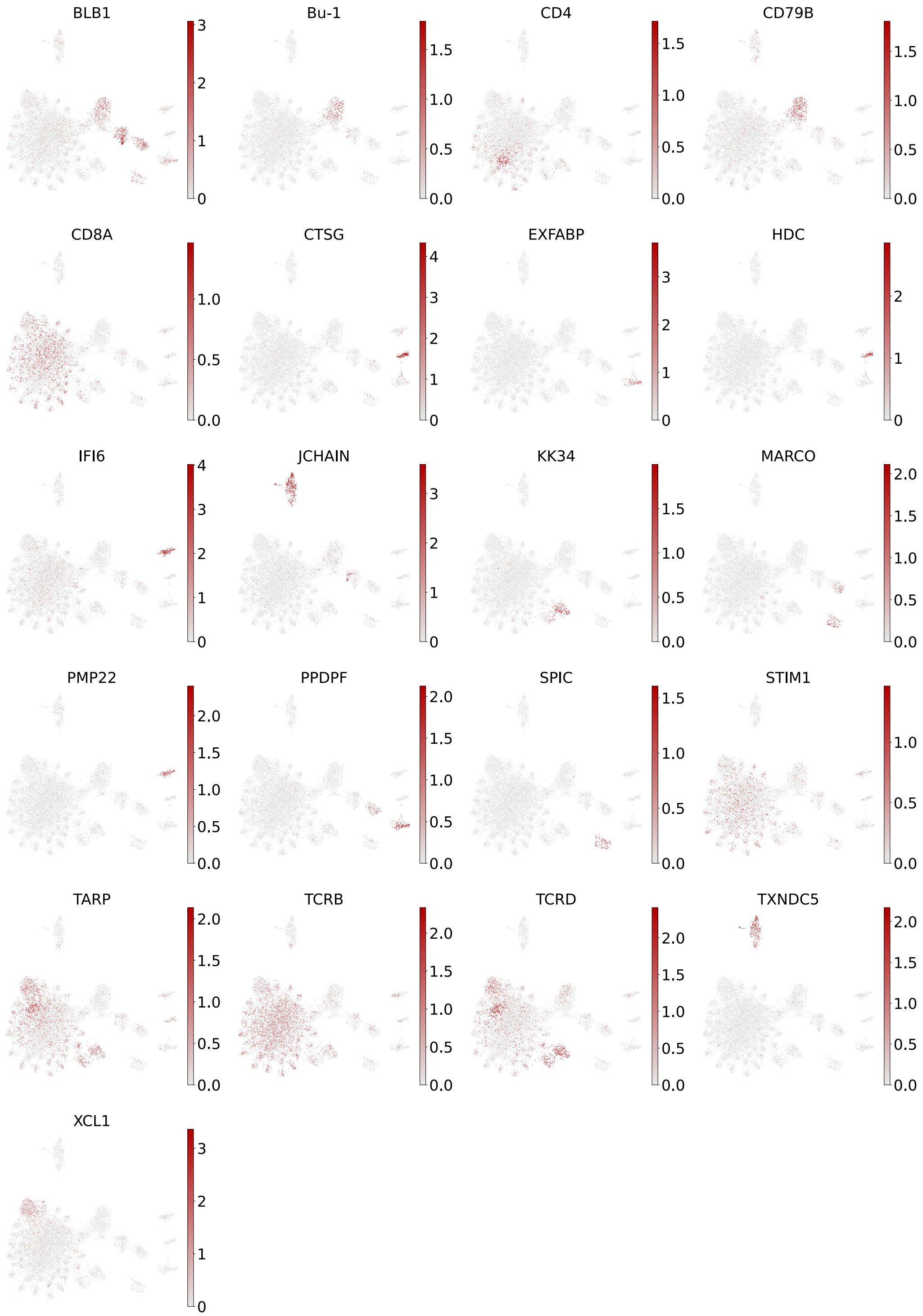

Supplement Figure 2. Expression of key marker genes by cell. Each plot is a UMAP projection of all cells with each cell colored based on expression of a given gene. Color scales are present to the right of each plot and differ based on the range of expression of the given gene. Each cell is colored on a spectrum based on the minimum and maximum cell expression of that gene to best visualize each gene's range of expression.

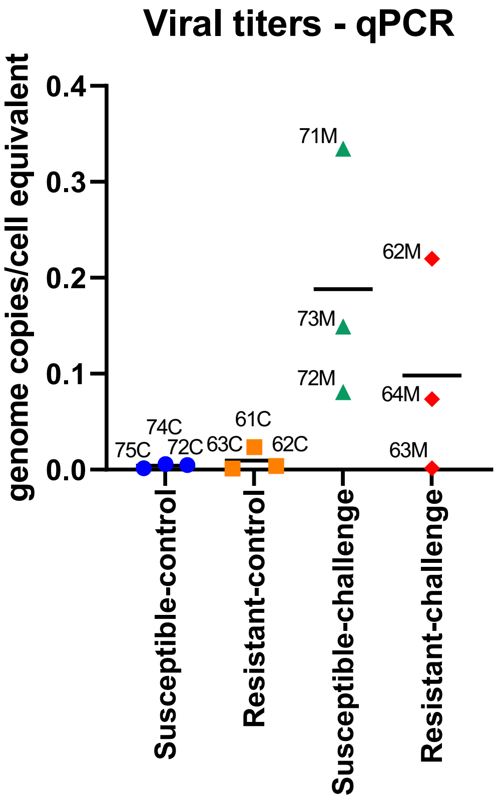

Supplement Figure 3. Quantitative PCR estimated MDV genome copies by line and infection state. Sample names are defined with first digit representing line 6 or 7, second digit representing the biological replicate, and the third character indicating whether the bird was infected with MDV (M) or an uninfected control (C). See methods for experimental details.
